# Supplementary material for: The mitochondrial calcium uniporter regulates breast cancer progression via HIF‐1α
Source: EMBO Mol Med. 2016 Apr 4;8(5):569–85. doi: 10.15252/emmm.201606255 (PMC4864890; doi:10.15252/emmm.201606255)
Supplement: Supplementary file 1 — Appendix [file EMMM-8-569-s001.pdf]

Table of contents

**Appendix Figure S1**
**Appendix Figure S2**
**Appendix Figure S3**
**Appendix Figure S4**
**Appendix Figure S5**
**Appendix Figure Legends**

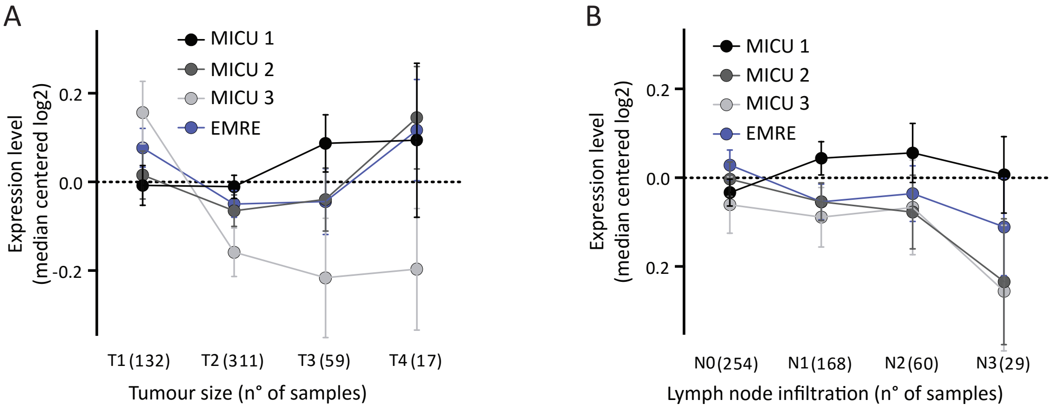

Appendix Figure S1, related to Figure 1

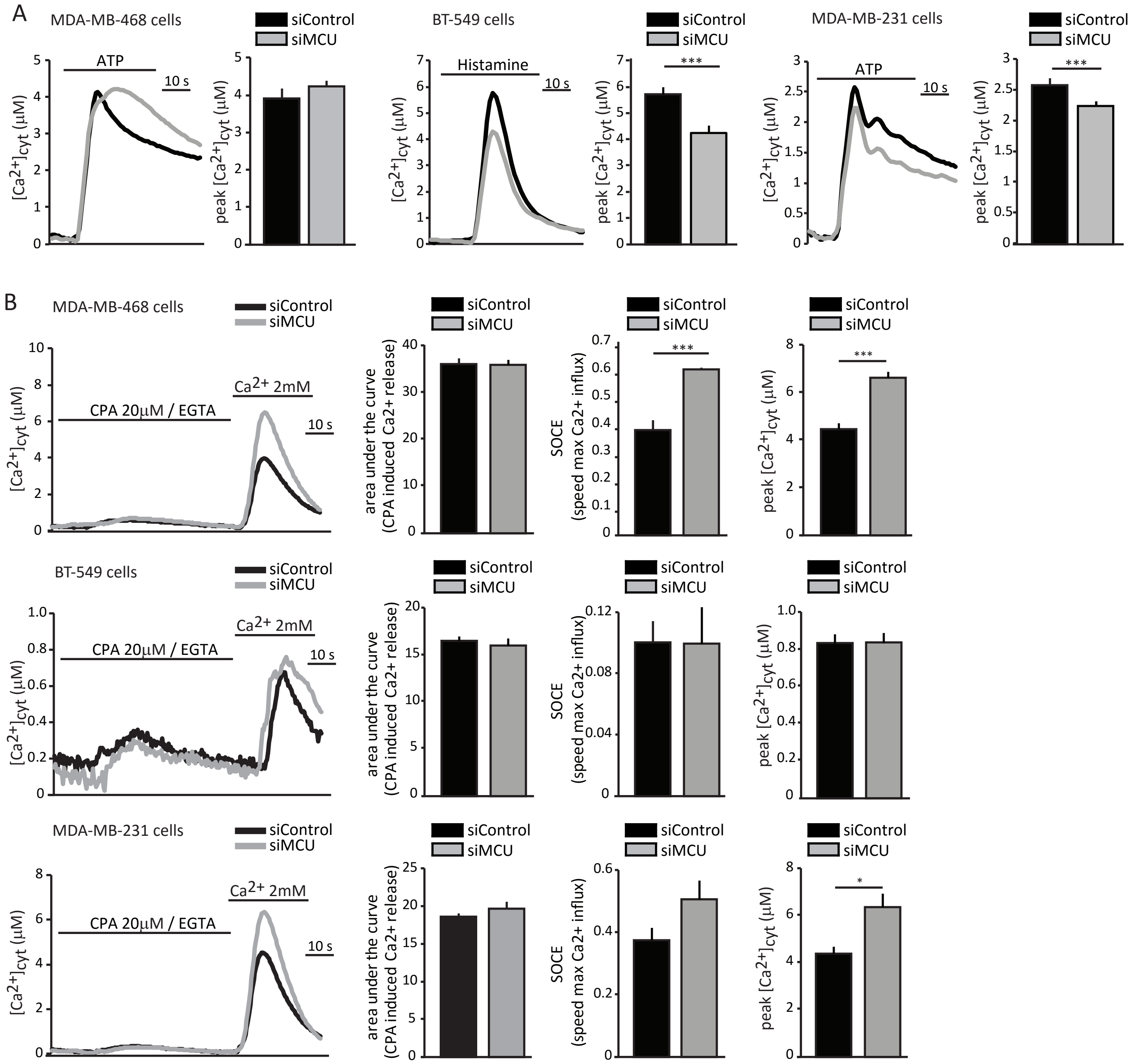

Appendix Figure S2, related to Figure 1 (continued on next page)

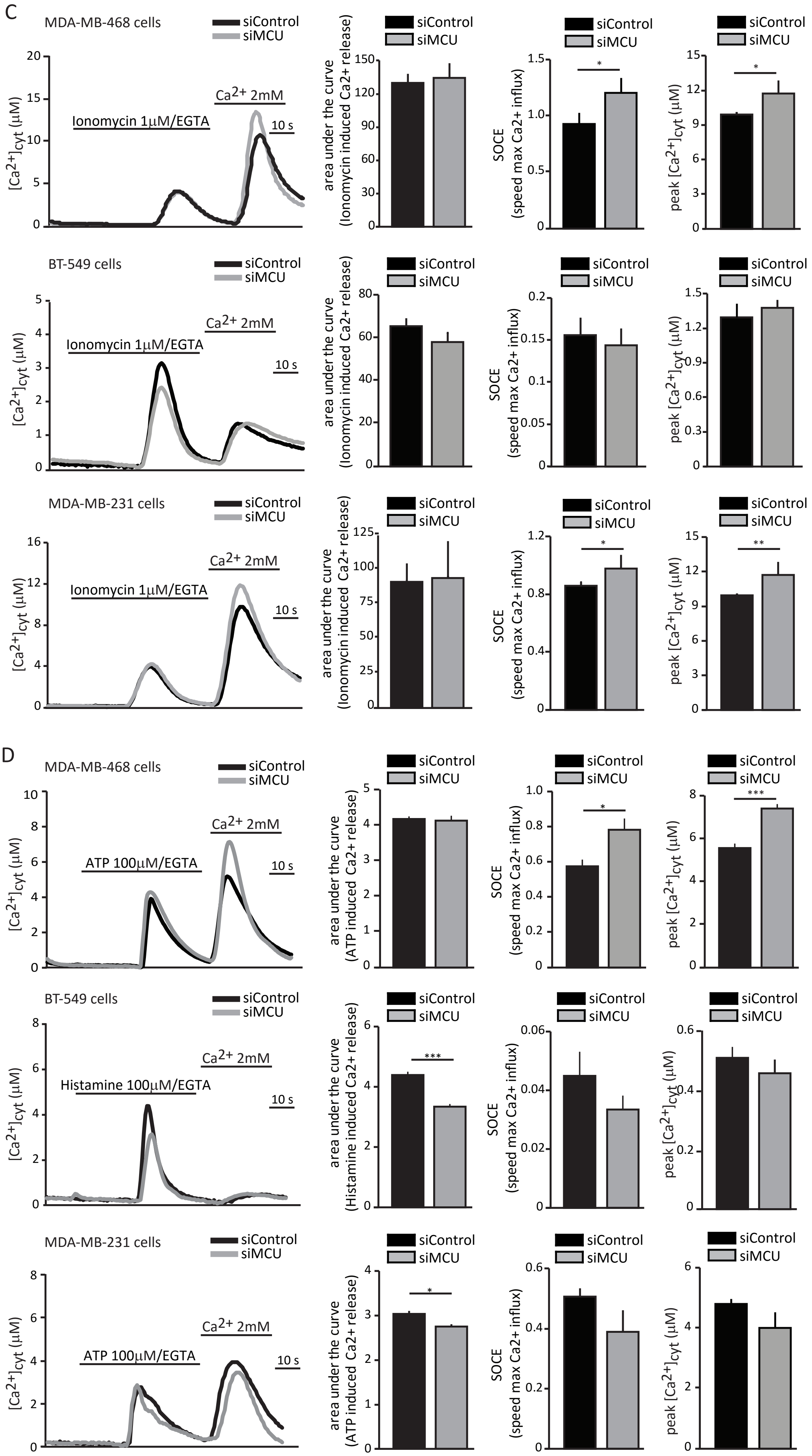

Appendix Figure S2, related to Figure 1

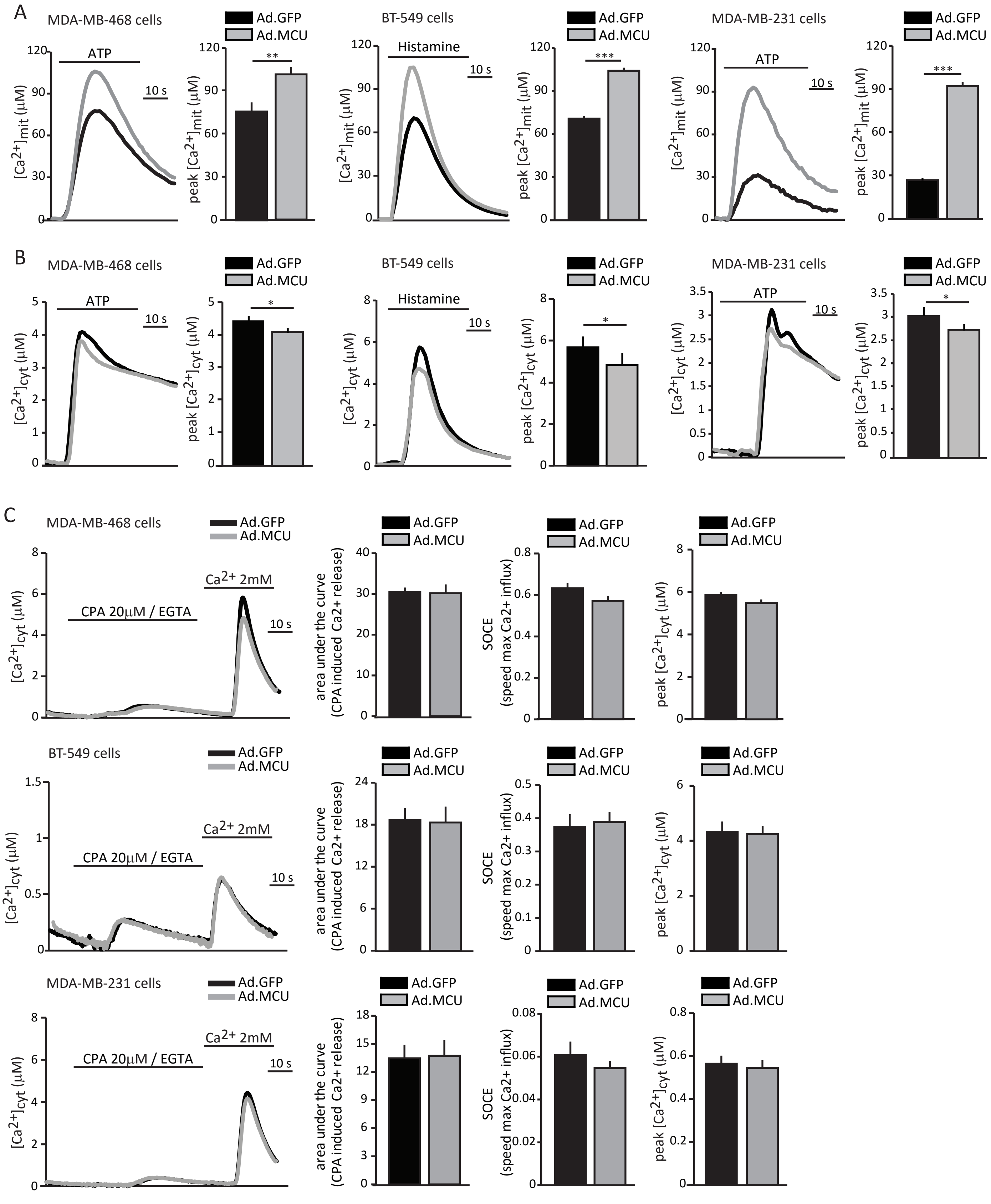

Appendix Figure S3, related to Figure 1 (continued on next page)

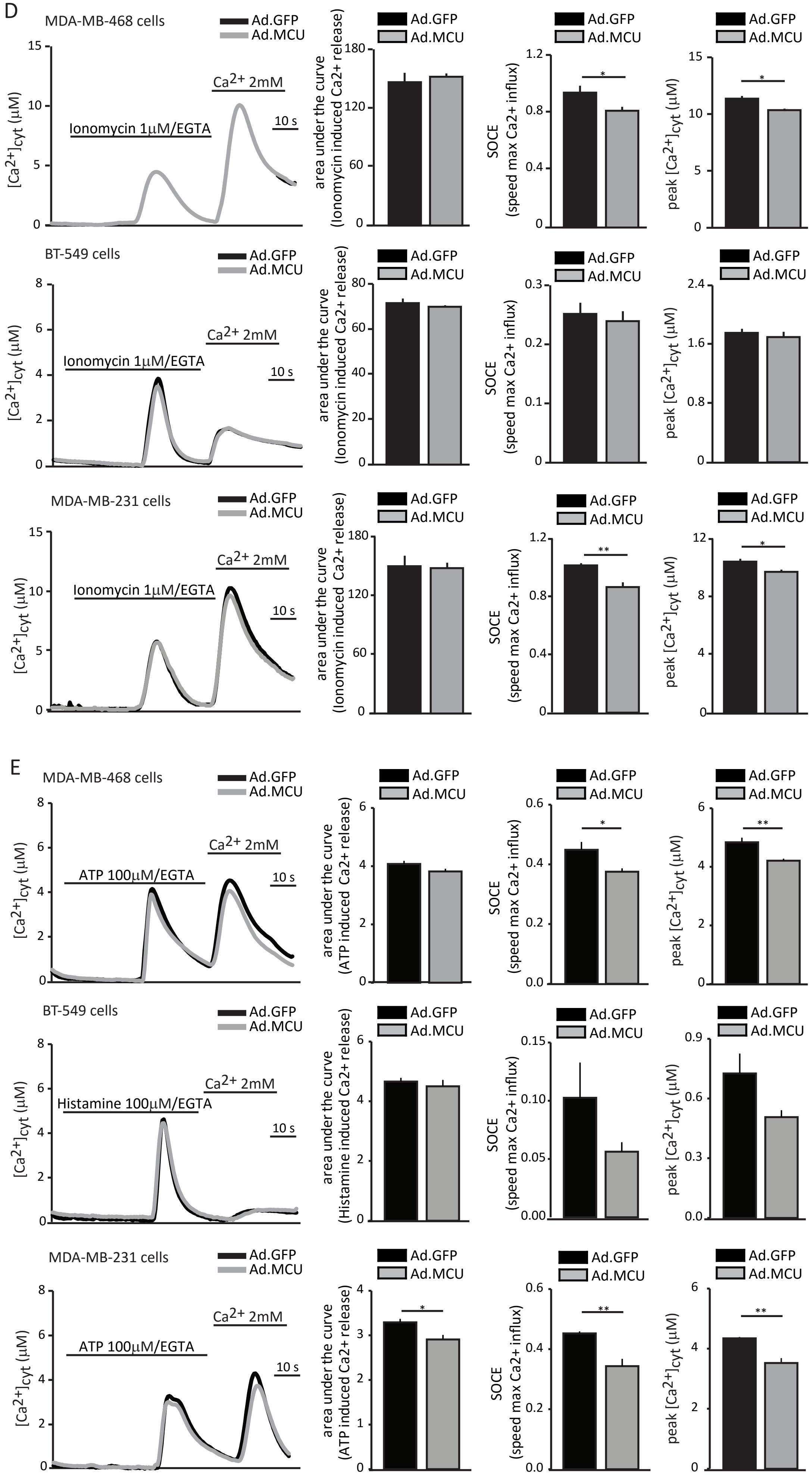

Appendix Figure S3, related to Figure 1

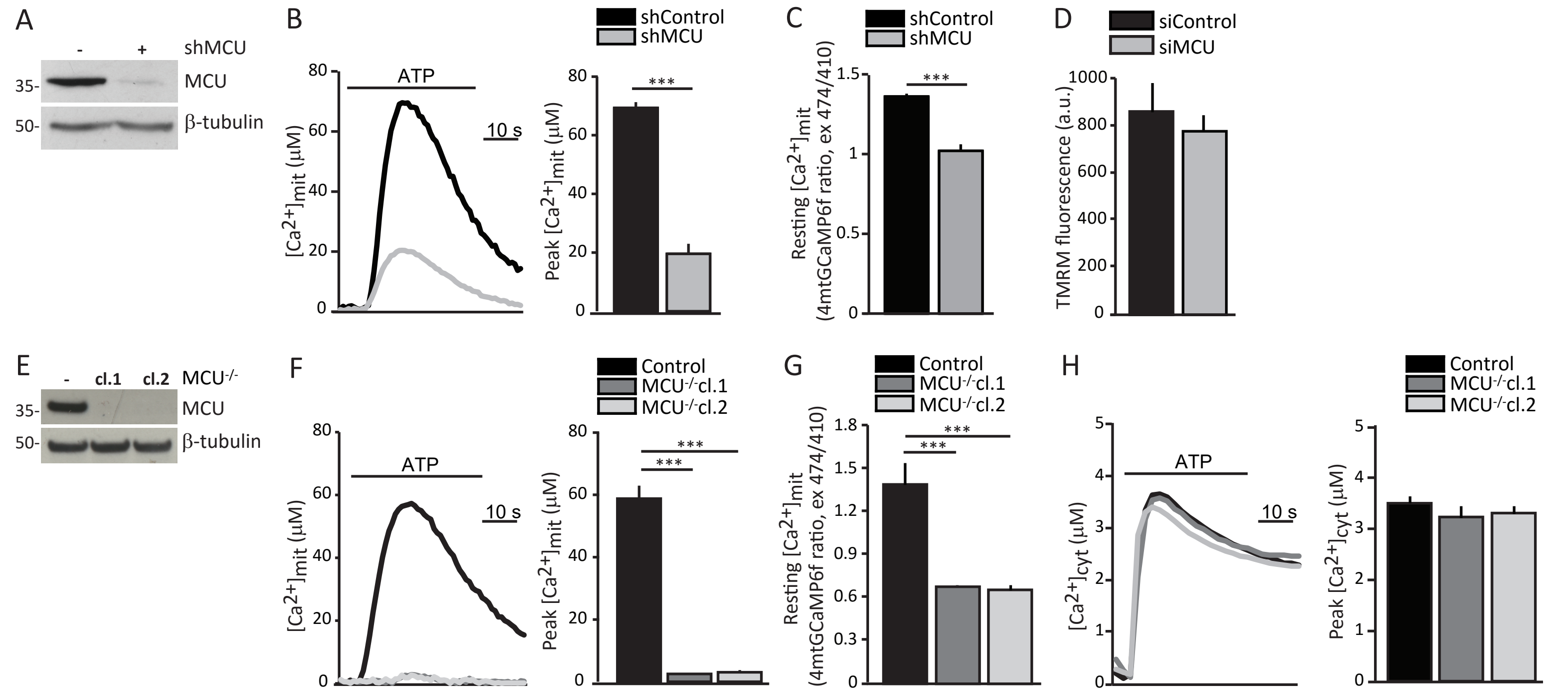

Appendix Figure S4, related to Figures 2 and 3

A

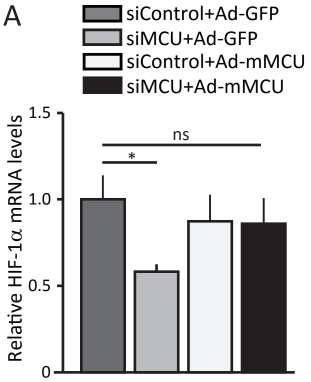

Appendix Figure S5, related to Figure 6

Appendix Figure Legends

Appendix Figure S1. Correlation of MCU complex component expression levels with breast cancer clinical stages

Median-centered log2 mRNA expression levels of MICUs1-3 and EMRE were collected from the TCGA breast cancer dataset (http://tcga-data.nci.nih.gov/docs/publications/brca_2012/). Data were plotted and analysed against tumour size (T1-T4) (**A**) and regional lymph node infiltration (N0-N3) (**B**) as described in Figure 1. Number of samples for each stage are shown in parentheses.

Appendix Figure S2. Cytosolic Ca^{2+} transients, SOCE, and ER Ca^{2+} content measurements in TNBC cell lines upon MCU silencing

A. MCU silencing decreases agonist-induced Ca^{2+}_{cyt} transients in BT-549 and MDA-MB-231 cell lines but not in MDA-MB-468. TNBC cells were transfected with siMCU or siControl. 48h later, Ca^{2+}_{cyt} transients upon agonist stimulation were measured (n=6). P-values: ***p<0.0001, ***p=0.0009.

B. MCU silencing increases SOCE in MDA-MB-231 and MDA-MB-468, but not in BT-549 cell lines. TNBCs cells were transfected with siMCU or siControl. 48h later, Ca^{2+} influx through SOCC, after CPA -mediated ER Ca^{2+} content depletion, was measured (n=6). P-values: (MDA-MB-468) ***p=0.0002, ***p=0.0002, (MDA-MB-231) *p=0.014.

C. MCU silencing increases SOCE in MDA-MB-231 and MDA-MB-468, but not in BT-549 cell lines. TNBCs cells were transfected with siMCU or siControl. 48h later, Ca^{2+} influx through SOCC, after Ionomycin-mediated ER Ca^{2+} content depletion, was measured (n=6). P-values: (MDA-MB-468) *p=0.0095, *p=0.0022, (MDA-MB-231) *p=0.03, **p=0.009.

D. MCU silencing increases SOCE in MDA-MB-468 but not in MDA-MB231 and BT-549 cell lines. TNBCs cells were transfected with siMCU or siControl. 48h later, Ca^{2+} influx through SOCC, after agonist-mediated ER Ca^{2+} content depletion, was measured (n=6). P-values: (MDA-MB-468) *p=0.017, ***p=0.0002, (BT-549) ***p=0.0001, (MDA-MB-231) *p=0.02.

In each panel, representative traces are shown (left), whereas ER Ca^{2+} content and SOCE are presented as mean ± SE (right). A two-tailed unpaired *t*-test was performed.

Appendix Figure S3. Cytosolic Ca^{2+} transients, SOCE, and ER Ca^{2+} content measurements in TNBC cell lines upon MCU overexpression

A. MCU overexpression increases agonist-induced Ca^{2+}_{mit} uptake in all three TNBC cell lines. TNBC cells were infected with Ad-mMCU or Ad-GFP. 48h later, Ca^{2+}_{mit} uptake upon agonist stimulation were measured (n=6). P-values: (MDA-MB-468) **p=0.0051, (BT-549) ***p<0.0001, (MDA-MB-231) ***p<0.0001.

B. MCU overexpression decreases agonist-induced Ca^{2+}_{cyt} transients in all three TNBC cell lines. TNBC cells were infected with Ad-mMCU or Ad-GFP. 48h later, Ca^{2+}_{cyt} transients upon agonist stimulation were measured (n=6). P-values: (MDA-MB-468) *p=0.011, (BT-549) *p<0.05, (MDA-MB-231) *p<0.03.

C-E. effects of MCU overexpression on SOCE. TNBC cells were infected with Ad-mMCU or Ad-GFP. 48h later, Ca^{2+} influx through SOCC, after depletion of ER Ca^{2+} content by CPA (C), ionomycin (D) or agonist treatment (E), was measured (n=6). P-values: (D) (MDA-MB-468) *p=0.04, *p=0.02, (MDA-MB-231) **p=0.0071, *p<0.03, (E) (MDA-MB-468) *p=0.04, **p=0.0069, *p=, (MDA-MB-231) *p=0.0169, **p<0.0017, **p=0.0015.

In each panel, representative traces are shown (left), whereas ER Ca^{2+} content and SOCE are presented as mean ± SE (right). A two-tailed unpaired *t*-test was performed.

Appendix Figure S4. Effects of stable MCU depletion on Ca^{2+} signalling

A. shMCU causes a sharp reduction in MCU expression. Western blot analysis of MCU expression in shMCU MDA-MB-231 cells.

B. Agonist-induced Ca^{2+}_{mit} uptake is decreased by shMCU. $[Ca^{2+}]_{mit}$ uptake upon ATP stimulation was measured (n=6). P-value: ***p<0.0001.

C. Resting Ca^{2+}_{mit} is decreased by shMCU. shMCU cells were transiently transfected with 4mtGCaMP6f. Resting Ca^{2+}_{mit} was measured. P-value: ***p<0.0001.

D. MCU silencing does not affect mitochondrial membrane potential. Cells were transfected with siMCU or siControl. 48 hours later, cells were loaded with tetramethylrhodamine ethyl ester (TMRE) and fluorescence was measured (n=6).

E. $MCU^{-/-}$ MDA-MB-231 clones were checked for depletion of MCU expression. Western blot analysis was performed on MCU-/- clones.

F. Agonist-induced Ca^{2+}_{mit} uptake is blunted in $MCU^{-/-}$ clones. $[Ca^{2+}]_{mit}$ uptake upon ATP stimulation was measured (n=8). P-values: ***p<0.0001.

G. Resting Ca^{2+}_{mit} is decreased in $MCU^{-/-}$ clones. $MCU^{-/-}$ cells were transiently transfected with 4mtGCaMP6f. Resting Ca^{2+}_{mit} was measured. P-values: ***p<0.0001.

H. Agonist-induced Ca^{2+}_{cyt} level is unaltered in $MCU^{-/-}$ clones. $[Ca^{2+}]_{cyt}$ transient upon ATP stimulation was measured (n=6).

In each panel, representative traces are shown (left), whereas Ca^{2+}_{mit} and Ca^{2+}_{cyt} content are presented as mean ± SE (right). A two-tailed unpaired *t*-test was performed.

Appendix Figure S5. Effects of MCU re-expression on HIF1α transcription

A. Expression of mouse MCU rescues HIF1α expression upon depletion by siMCU. MDA-MB-231 cells transfected with siMCU and were infected with an Ad-mMCU. mRNA expression was quantified by Real-Time PCR.
